# Activation of AMPK by the Putative Dietary Restriction Mimetic Metformin Is Insufficient to Extend Lifespan in *Drosophila*

**DOI:** 10.1371/journal.pone.0047699

**Published:** 2012-10-16

**Authors:** Cathy Slack, Andrea Foley, Linda Partridge

**Affiliations:** 1 Institute of Healthy Ageing, Department of Genetics Evolution and Environment, University College London, London, United Kingdom; 2 Max Planck Institute for Biology of Ageing, ZMMK Forshungsgebaude, Koln, Germany; University of Houston, United States of America

## Abstract

The biguanide drug, metformin, commonly used to treat type-2 diabetes, has been shown to extend lifespan and reduce fecundity in *C. elegans* through a dietary restriction-like mechanism via the AMP-activated protein kinase (AMPK) and the AMPK-activating kinase, LKB1. We have investigated whether the longevity-promoting effects of metformin are evolutionarily conserved using the fruit fly, *Drosophila melanogaster.* We show here that while feeding metformin to adult *Drosophila* resulted in a robust activation of AMPK and reduced lipid stores, it did not increase lifespan in either male or female flies. In fact, we found that when administered at high concentrations, metformin is toxic to flies. Furthermore, no decreases in female fecundity were observed except at the most toxic dose. Analysis of intestinal physiology after metformin treatment suggests that these deleterious effects may result from disruptions to intestinal fluid homeostasis. Thus, metformin appears to have evolutionarily conserved effects on metabolism but not on fecundity or lifespan.

## Introduction

Dietary restriction (DR), defined as a reduction in caloric intake that is not accompanied by malnutrition, increases lifespan in all species tested so far including primates [1], [2], making it the most robust longevity-assurance mechanism identified to date. In addition to its effects on lifespan, DR has also been shown to slow the progression of age-related functional decline and delay the onset of several age-related diseases in laboratory animals (reviewed in [3]). Data from dietary restriction studies in humans has revealed several metabolic benefits of DR similar to those observed in model organisms [4], [5] suggesting that DR may also be beneficial to health and longevity in humans. Long-term DR in humans, however, is impractical as the majority of people are unwilling to significantly restrict their food intake over a long period of time. Thus, pharmacological interventions that recapitulate the health benefits of DR without necessitating a reduction in food intake (so called DR-mimetics) could provide an attractive method of improving health and well-being in older people.

One such putative DR-mimetic is the biguanide drug, metformin, widely prescribed as a treatment for type-2 diabetes. Metformin has been shown to induce a similar, overlapping transcriptional profile to both short-term and long-term DR especially in metabolic transcripts [6] suggesting that these interventions modulate similar downstream pathways. In support of this, metformin induces physiological phenotypes similar to those produced by DR. Thus, metformin lowers blood glucose, predominantly by decreasing its production in the liver [7], increases insulin-dependent glucose uptake in peripheral tissues [8], lowers circulating insulin levels [9] and promotes fatty acid metabolism [10], all physiological hallmarks of DR [3]. In addition, metformin has been shown to delay disease progression and improve survival in several rodent models that are prone to cancer or other diseases [11], [12], [13], [14], . Even in diabetic human patients, metformin treatment is associated with lowered cancer incidence and increased survival, although these effects may be primarily mediated by increased weight loss in obese diabetic patients [18]. However, the effects of metformin on lifespan in a heterogeneous long-lived population are less clear and the results from laboratory studies using rodents are confounded by several experimental factors including the use of short-lived or disease model strains [11], [13], [15], reductions in food consumption and/or body weight in metformin-treated animals [16], absence of metabolic phenotypes normally induced by metformin [19], lack of a positive control within the experiment (for example, a DR group) [17] or absence of lifespan extension in the positive control group [19].

Recently, a comprehensive study in *C. elegans* has shown that metformin increases the lifespan of wild-type worms and produces several DR-like phenotypes in otherwise fully-fed animals, including reduced fecundity and lowered fat stores [20]. These effects of metformin were not additive with a genetic model of DR in worms, the *eat-2* mutation, suggesting that metformin increases lifespan via a DR-related mechanism in worms [20].

The molecular targets of metformin are still not well characterised, although one candidate is the AMP-activated protein kinase (AMPK). AMPK is a key nutrient sensor that plays an important role in metabolism and the regulation of whole body energy balance [21], [22]. AMPK is activated by changes in the AMP/ATP ratio and upon activation, induces catabolic pathways to restore ATP levels by promoting glycolysis and fatty acid oxidation as well as increasing mitochondrial biogenesis and the metabolism of mitochondrial substrates [23]. In mammals, AMPK and the AMPK-activating kinase, LKB1, are also activated by metformin [21], [24] and this activation is required for several of the metabolic effects of metformin including fatty acid oxidation and the inhibition of glucose production by liver cells [21], [25]. The mechanism by which metformin increases AMPK activity remains unclear, although neither AMPK nor LKB1 are thought to be direct targets of metformin. More likely, metformin activates AMPK by increasing the amount of cytosolic AMP as metformin inhibits complex I of the mitochondrial electron transport chain [26] and inhibits AMP deaminase [27] thus decreasing ATP production.

In addition to metabolic regulation, AMPK also plays an important role in the regulation of lifespan. In *Drosophila*, ubiquitous reduction of AMPK activity [28] or inhibition of AMPK specifically in muscle is sufficient to decrease lifespan [29] while over-expression of LKB1 also promotes longevity in flies [30]. In *C elegans*, genetic deletion of the AMPK catalytic subunit, *aak-2*, decreases lifespan while over-expression of *aak-2* increases lifespan [31] and several DR protocols require *aak-2* to promote DR-mediated lifespan extension [32], [33]. Furthermore, both *aak-2* and *lkb-1* are required in worms to mediate the lifespan effects of metformin [20]. Thus, in worms AMPK is an important mediator of the beneficial effects of both DR and metformin.

In this study, we have examined whether the beneficial effects of metformin treatment on the lifespan of otherwise fully fed animals are evolutionary conserved using the fruit fly, *Drosophila melanogaster*. We demonstrate that while oral administration of metformin to adult flies activates the fly AMPK, this does not lead to an increase in lifespan. In fact, we found that metformin is toxic to flies in a dose-dependent manner. Unlike in worms, metformin treatment in flies did not delay or reduce reproduction except at the most toxic dose. We did, however, find that metformin reduces fat stores in flies even at a moderate dosage. The toxicity of metformin in flies may be mediated via perturbations in intestinal homeostasis as flies treated with high concentrations of metformin produce more concentrated faecal deposits indicative of intestinal fluid imbalance. Taken together, our data suggest that metformin treatment has overlapping effects on AMPK activation and lipid metabolism in worms and flies but that the beneficial effects of metformin on longevity are not conserved between these two species.

## Results

### Activation of Drosophila AMPK after Ingestion of Metformin

**Figure 1:**
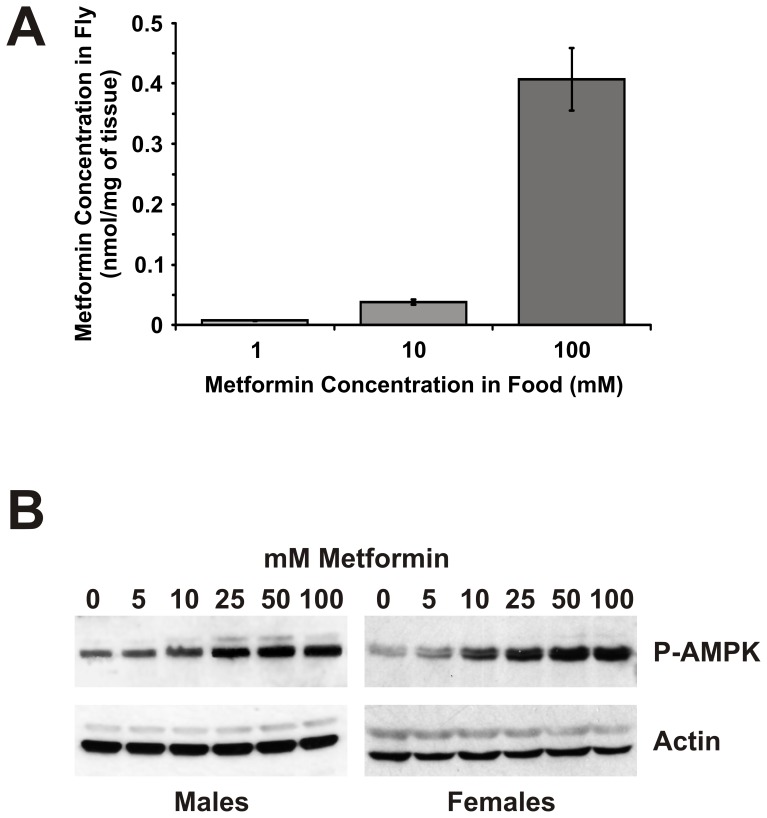
Metformin treatment of adult *Drosophila* activates AMPK. A. Mass spectrometric determination of metformin concentration in whole fly extracts. Female flies were sampled after 7 days of metformin treatment at concentrations of 1, 10 and 100 mM. Before sampling, flies were incubated in the absence of metformin for 5 hours to allow for gut emptying. A dose-dependent increase in metformin accumulation in fly tissues was observed. Data are represented as the mean of three independent replicate samples ± SEM. **B.** Western blot analysis of phospho-Thr172-AMPK expression in whole-fly protein extracts. Flies were sampled after 7 days of metformin treatment at concentrations of 0, 5, 10, 25, 50 and 100 mM. A dose-dependent increase in phospho-Thr172-AMPK levels was observed. Actin was used as a loading control.

We first assessed the efficacy of oral administration of metformin in *Drosophila* by measuring its accumulation in fly tissues. Adult flies were fed increasing concentrations of metformin for 7 days and fly extracts were prepared after the gut was cleared of ingested food. We could readily detect metformin in tissue extracts by mass spectrometric analysis even at the lowest administered dose of 1 mM (Figure 1A). Moreover, the concentration of metformin in fly tissue extracts increased with increasing concentration in the food so with 10 mM and 100 mM metformin in the food the concentration of metformin in fly tissue extracts increased to 0.04±0.004 nmol/mg and 0.4±0.05 nmol/mg, respectively (Figure 1A). This is comparable to the tissue concentration of metformin in mice after oral administration which is in the range of 0.012 to 2.29 nmol/mg of tissue depending on the tissue type [34]. Thus, metformin appears to be efficiently absorbed from the fly gut after ingestion and accumulates in the body tissues.

To determine if oral administration of metformin activates AMPK in *Drosophila*, we measured phosphorylation of the catalytic subunit of AMPK at Thr172 which is required for AMPK activation. Western blot analysis of whole-fly protein extracts using a phospho-specific antibody revealed a dose-dependent increase in phospho-Thr172-AMPK levels after metformin treatment (Figure 1B) confirming that oral ingestion of metformin activates *Drosophila* AMPK in vivo.

### Metformin Treatment does not Extend Lifespan in Drosophila

**Figure 2:**
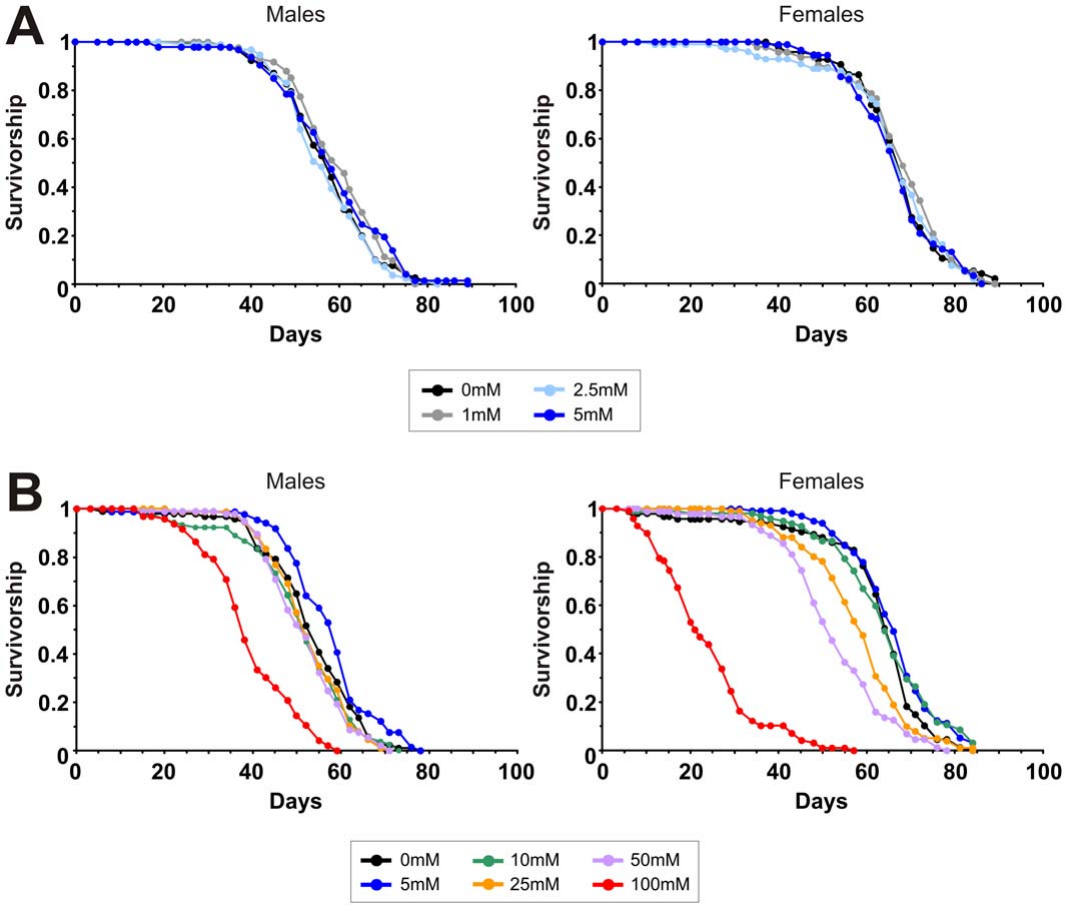
Metformin does not increase lifespan in *Drosophila.* A. Survival curves of wild-type (*Dahomey*) males and females maintained on food containing no metformin or final concentrations of 1 mM, 2.5 mM or 5 mM metformin. No significant differences in survival were observed between metformin treated flies and non-treated controls by the Log-rank test. For males, median survival times on 0 mM, 1 mM, 2.5 mM and 5 mM metformin were 57 (n = 96), 59 (n = 96), 55 (n = 97) and 57 (n = 96) days, respectively. For females, median survival times were 67 days for all conditions (0 mM n = 96, 1 mM n = 96, 2.5 mM n = 99 and 5 mM n = 91). **B.** Survival curves of wild-type (*Dahomey*) males and females maintained on food containing no metformin or final concentrations of 5 mM, 10 mM, 25 mM, 50 mM or 100 mM metformin. The survival curves for males maintained on 0 mM, 5 mM, 10 mM, 25 mM and 50 mM are not significantly different, while males maintained on 100 mM metformin were significantly shorter lived than non-treated controls (*P*<0.0001 by the Log-rank test). Median survival times for males on 0 mM, 5 mM, 10 mM, 25 mM, 50 mM and 100 mM metformin were 54 (n = 97), 58 (n = 91), 51 (n = 93), 51 (n = 93), 51 (n = 98) and 37 (n = 99) days, respectively. The survival curves for females maintained on 0 mM, 5 mM and 10 mM metformin are not significantly different. Females maintained on 25 mM, 50 mM and 100 mM metformin were significantly shorter lived than non-treated controls (*P*<0.001 by the Log-rank test). Median survival times for females on 0 mM, 5 mM, 10 mM, 25 mM, 50 mM and 100 mM metformin were 65 (n = 96), 65 (n = 100), 63 (n = 98), 58 (n = 101), 51 (n = 91) and 22 (n = 99) days respectively.

We next investigated the effects of metformin on *Drosophila* lifespan when administered at different concentrations. No effect on survival was observed for male flies maintained on food containing 1 mM, 2.5 mM, 5 mM, 10 mM, 25 mM or 50 mM metformin, with a significant decrease in survival at 100 mM metformin (Figure 2A and 2B). For female flies, again no effect on survival was observed at 1 mM, 2.5 mM, 5 mM or 10 mM metformin, while increasing the concentration of metformin above 10 mM resulted in a dose-dependent decrease in lifespan (Figure 2A and 2B). Thus, metformin treatment in *Drosophila* does not appear to offer any benefits for survival and is actually toxic at higher doses.

**Figure 3:**
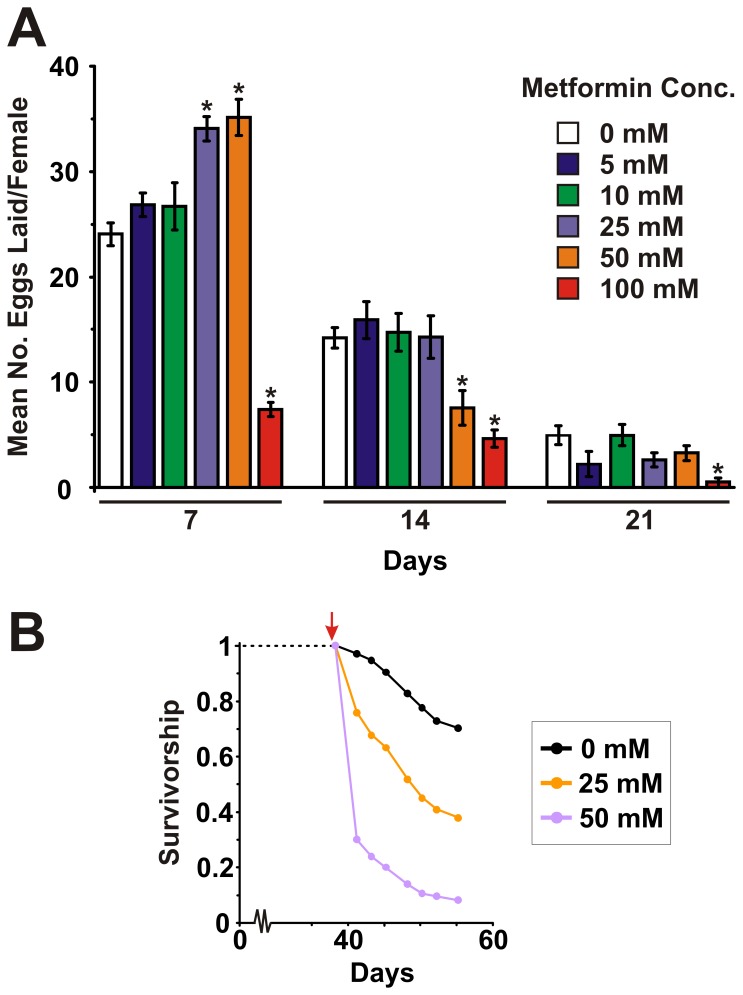
Effects of metformin on female egg-laying and post-reproductive survival. (**A**) Egg-laying profiles of wild-type females treated with 0 mM, 5 mM, 10 mM, 25 mM, 50 mM and 100 mM metformin. Eggs were counted from 10 vials per treatment (10 females per vial) over a 24 hour period after 7, 14 and 21 days of metformin treatment. Data are shown as means ±SEM. * denotes statistically significant difference (*P*<0.05). No significant differences were observed in egg-laying between females on 0 mM, 5 mM and 10 mM metformin at any time point. After 7 days of treatment, females on 25 mM and 50 mM metformin laid significantly more eggs than untreated controls. After 14 days of treatment, females on 50 mM laid significantly fewer eggs than untreated controls. Females on 100 mM metformin laid significantly fewer eggs than untreated controls at all time points. (**B**) Survival curves for post-reproductive wild-type females maintained on food containing no metformin or final concentrations 25 mM and 50 mM metformin. Flies (n = 250 for each concentration) were switched onto food containing metformin at 39 days of age (red arrow). Females maintained on 25 mM or 50 mM showed reduced survival compared to flies maintained in the absence of metformin (*P*<0.001 by the Log-rank test).

In *C. elegans*, metformin not only increases lifespan but also causes both a reduction and delay in reproduction [20]. We therefore examined the effects of metformin treatment on female egg-laying. Females fed with 5 mM and 10 mM metformin did not show any differences in egg-laying compared to untreated control females. After 7 days of treatment, females on 25 mM and 50 mM metformin laid significantly more eggs than untreated controls but after 14 days of treatment, egg-laying in females on 25 mM metformin was comparable to untreated controls while females on 50 mM laid significantly fewer eggs than untreated controls. Females on 100 mM metformin laid significantly fewer eggs than untreated controls at all time points. No delay in reproduction was observed for any concentration of metformin used (Figure 3A).

It is possible that the increased egg-laying observed for females treated with 25 mM and 50 mM metformin may contribute to their decreased survival because reproduction and somatic maintenance may compete for nutritional resources [35]. To test this, we maintained females in the absence of metformin until they had reached a post-reproductive age (39 days) and then switched them onto food containing 0 mM, 25 mM or 50 mM metformin and monitored their survival. We found that even in the absence of egg-laying, feeding flies with either 25 mM or 50 mM metformin decreased their survival (Figure 3B).

### Metformin Induces a Dose-Dependent Reduction in Fat Stores

**Figure 4:**
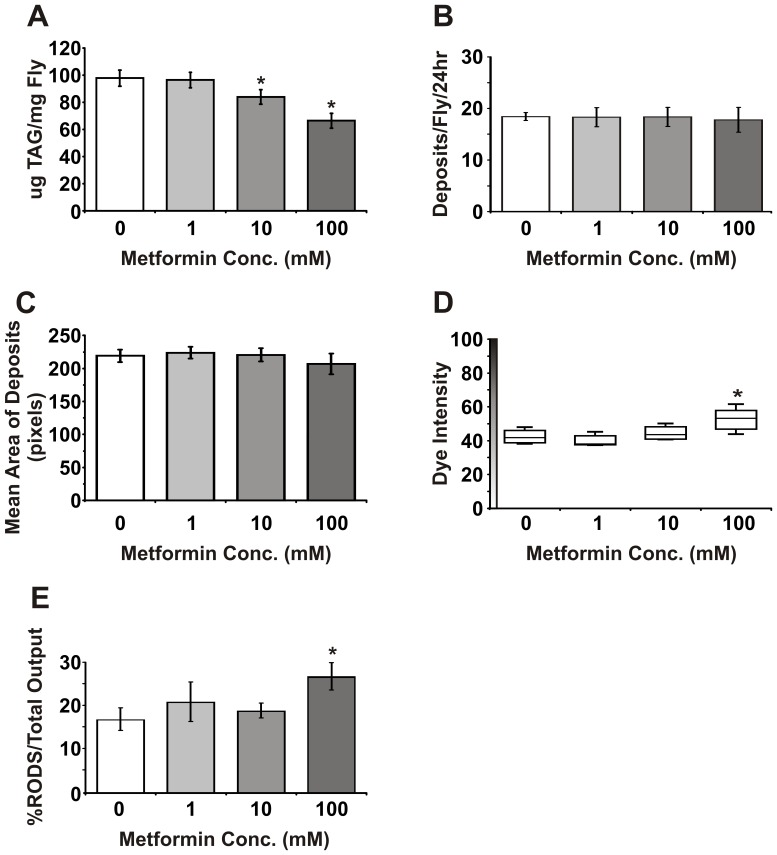
Metformin reduces lipid stores and causes intestinal fluid imbalance. (**A**) Quantitation of triacylglycerides (TAGs) in flies treated with 0 mM, 1 mM, 10 mM and 100 mM metformin for 7 days. TAG levels decrease with increasing metformin concentration with significantly lower levels in the 10 mM and 100 mM metformin groups compared to untreated controls (*P*<0.05, n = 10 (2 flies per replicate)). (**B**) Metformin treatment of female flies does not affect the number of fecal deposits produced per fly over a 24 hour period (*P>0.05*, Wilcoxen test, n = 5 (5 flies per replicate)). (**C**) Metformin treatment of female flies does not affect the size of fecal deposits as measured by the mean area of deposits (*P>0.05*, Wilcoxen test, n = 5 (5 flies per replicate)). (**D**) Female flies fed with 100 mM metformin produce more concentrated fecal deposits as measured by increased average dye intensity (*P*<0.05, Wilcoxen test, n = 5 (5 flies per replicate)). (**E**) Female flies fed with 100 mM metformin produce more RODs as a percentage of their total excreta output (*P*<0.05, Wilcoxen test, n = 5 (5 flies per replicate)).

In mammals, activation of AMPK by metformin induces fatty acid oxidation and inhibits lipogenesis, reducing the levels of stored lipids. Lipid stores are also decreased in worms raised in the presence of metformin [20]. In *Drosophila*, lipids are primarily stored as triaceylglycerides (TAGs) in the fat body and after 7 days of treatment, TAG levels were significantly reduced in females fed with 10 mM or 100 mM metformin compared to untreated controls (Figure 4A). Thus, metformin also has dose-dependent effects on fat metabolism in *Drosophila*.

### Metformin Treatment Disrupts Intestinal Fluid Homeostasis

A common side-effect of metformin treatment in diabetic patients is gastrointestinal upset. We therefore used a recently developed assay to examine the effects of metformin on intestinal physiology in *Drosophila* by examining fly excreta [36]. After 7 days of treatment with 0 mM, 1 mM, 10 mM and 100 mM metformin, Bromophenol blue was added to the food and then the resulting coloured excreta were collected for 24 hours. There were no obvious differences in the number or size of excreta deposits between metformin treated flies and untreated controls (Figure 4B and 4C). However, the intensity of the Bromophenol blue dye within the deposits of flies treated with 100 mM metformin was significantly higher than untreated controls (Figure 4D) suggesting that the faecal output of these flies is more concentrated. In addition, flies fed with 100 mM metformin excrete a greater proportion of concentrated oblong deposits called RODS or reproductive oblong deposits [36] (Figure 4E). These data suggest that treatment with high concentrations of metformin lead to fluid imbalances in the fly intestine resulting in the production of more concentrated faecal deposits.

## Discussion

In this study, we have examined whether the biguanide drug, metformin, functions as a dietary restriction (DR)-mimetic in *Drosophila*. In flies, DR is usually induced by dilution of dietary yeast while maintaining constant dietary sucrose [37], [38]. Under these conditions, DR increases both median and maximum lifespan and, in females, reduces fecundity [37], [38]. Despite testing a wide range of concentrations, we did not observe any beneficial effects of metformin treatment on lifespan in either male of female flies. Instead, metformin reduced survival in *Drosophila* in a dose-dependent manner. Our results are consistent with a previous study which also found no statistically significant effect of metformin treatment on mortality rate in *Drosophila* although only one concentration of metformin was used and the efficacy of this metformin dose was not tested [39]. We also saw no reduction in female egg-laying except at the most toxic dose, and at intermediate concentrations, metformin actually increased egg-laying at least at young ages. This increase in egg-laying was not, however, responsible for the observed decrease in survival because metformin still decreased survival when administered at post-reproductive ages.

Mass spectrometric analysis of metformin concentration in fly tissues confirmed that the drug was readily absorbed from the gut and we observed robust activation of the fly AMPK, a downstream target which is activated by metformin treatment in mammals and is required for several of its metabolic effects [21]. In worms, metformin-induced lifespan extension requires the activity of AMPK and its upstream activating kinase, LKB1 [20]. However, we have shown in this study that AMPK activation by metformin was not sufficient to extend lifespan in flies. AMPK is not the only downstream target of metformin. For example, metformin inhibits the target of rapamycin (TOR) kinase independently of AMPK in *Drosophila* [40]. It is therefore possible that metformin has deleterious AMPK-independent effects in flies that mask any beneficial effects of AMPK activation on lifespan. Hence, we cannot exclude the possibility that AMPK activation via alternative mechanisms may increase lifespan in flies. For example, recent evidence suggests that over-expression of the AMPK-activating kinase, LKB1, increases survival in *Drosophila* [30], although whether this effect is dependent on increased AMPK activity has yet to be ascertained.

Although the beneficial effects of metformin on lifespan do not appear to be evolutionarily conserved in *Drosophila*, we did observe conserved effects on fat metabolism in flies. In worms, metformin treatment is associated with reduced fat stores [20]. Similarly, in mammals metformin induces fatty acid oxidation and inhibits lipogenesis thereby reducing lipid stores [10], [41]. In *Drosophila* lipids are primarily stored as triaceylglycerides (TAGs) in the fat body, the insect equivalent of the mammalian liver and white adipose tissue. We have shown that metformin reduces TAG levels and interestingly, this reduction in lipid stores was observed at a non-toxic concentration of metformin at which both survival and female egg-laying were unaffected. It is therefore possible to triage the metabolic effects of metformin on fat metabolism from its deleterious effects on survival by simply reducing the dosage.

The minimum dose of metformin used in our experiments was 1 mM and this concentration produced no obvious physiological effects. For example, survival, female egg-laying, TAG levels and intestinal physiology in flies treated with 1 mM metformin were all comparable to untreated controls. Furthermore, AMPK activation is only apparent at doses above 5 mM. It is therefore unlikely that the optimal metformin dose for lifespan is lower than that tested.

The toxic effects of high concentrations of metformin in *Drosophila* could be mediated via several mechanisms. In *C. elegans,* metformin treatment at doses that extended lifespan in wild-type worms was found to be deleterious in combination with the *eat-2* mutation, possibly as a result of excessive metabolic down-regulation and induction of a starvation-like phenotype [20]. High levels of AMPK activity in metformin-treated flies may similarly reduce metabolic rates to such an extent as to induce analogous starvation-like phenotypes resulting in reduced survival and decreased egg-production. Flies exposed to high doses of metformin also showed several features of intestinal fluid imbalance including the production of more concentrated excreta, used as a method to preserve water in insects [36]. Interestingly, in several *Drosophila* species lowered levels of lipogenic stores correlate with reduced tolerance to desiccation [42]. The loss of lipid stores observed with high doses of metformin may therefore make flies more susceptible to fluid loss thereby decreasing their survival. At intermediate metformin concentrations we still observed a decrease in survival but without an associated decrease in egg production and so in these flies, mechanisms other than starvation or fluid imbalance must mediate metformin toxicity. In humans, the most potentially serious side-effect of metformin treatment is lactic acidosis [43] and so it is possible that acidosis in *Drosophila* contributes to the reduced survival of metformin treated flies.

Dietary restriction remains the most public intervention that can extend lifespan. Studies from model organisms have implicated several genetic pathways that regulate the DR response including the TOR pathway [44], [45] and insulin/insulin-like growth factor signalling [46], [47], [48] yet the precise molecular mechanisms whereby DR promotes survival remain elusive. It is also unclear whether the effects of DR on lifespan in different organisms are mediated via the same mechanism or have arisen as a result of convergent evolution. Pharmacological interventions that mimic the effects of DR in one model organism may therefore prove ineffectual in another. Our data would suggest that while metformin appears to function as a DR-mimetic in worms, its beneficial effects on survival do not translate across to *Drosophila*. In mammals, the effects of metformin on lifespan are yet to be tested in a long-lived outbred strain, using a range of concentrations, preferably alongside a DR group, and with sufficient data on food intake and body weight to exclude possible DR effects (reviewed in [49]). Therefore, the extent to which metformin functions as a DR-mimetic to benefit mammalian and importantly human lifespan remains to be seen.

## Materials and Methods

### Drosophila Culture

The wild-type stock *Dahomey* was collected in 1970 in Dahomey (now Benin) and has since been maintained in large population cages with overlapping generations on a 12L:12D cycle at 25°C. *Dahomey* is positive for the endosymbiont, *Wolbachia pipientis*. Stocks were maintained and all experiments were conducted at 25°C on a 12 h:12 h light:dark cycle at constant humidity using standard sugar/yeast/agar (SYA) medium [38]. For all experiments, flies were reared at standard larval density and eclosing adults were collected over a 12 hour period. Flies were mated for 48 hours before sorting into single sexes.

### Metformin Treatment

Metformin (Sigma) was added directly to SYA food from a 1M aqueous stock to give final concentrations of 1, 2.5, 5, 10, 25, 50 and 100 mM. For control food (0 mM) water alone was added.

### Lifespan Assays

For lifespan experiments, flies were maintained in vials at a density of 10 flies per vial on standard SYA medium. Flies were transferred to new vials three times per week and the number of deaths counted.

### Female Fecundity Assays

Female flies were housed with males for 48 hours post-eclosion and then separated into vials at a density of 10 females per vial. Eggs were collected over two 24-hour periods per week for 4 weeks. The number of eggs laid per vial at each time point was counted.

### Western Blotting

40 µg of total protein were resolved on 10% Tris-Glycine-SDS gels. Proteins were transferred to nitrocellulose membranes and probed for phospho-Thr172-AMPK (1∶1000; Cell Signaling) and actin (1∶5000; Sigma). Secondary antibodies conjugated to HRP were purchased from Biorad.

### Triacylglyceride (TAG) Measurements

Flies were homogenised in 0.05% Tween-20 and TAG levels were measured using the Triglyceride Infinity Reagent (ThermoScientific) and triglyceride standards. TAG levels were normalised to body weight.

### Mass Spectrometric Determination of Metformin Concentration in Flies

Flies were homogenised in phosphate buffered saline and all samples were spiked with 7.3 nM phenformin (Sigma) as an internal standard. For the standard curve, non-treated flies were homogenised and then samples spiked with metformin resulting in final concentrations of 5–80 ng/ml. Proteins were precipitated by the addition of acetonitrile. Mass spectrometric analysis of metformin concentration was performed by Dr Carolyn Hyde of the Scientific Support Services, Wolfson Institute for Biomedical Research, University College London using Shimadzu LC-MS-ITToF.

### Fly Defecation Assay

Analysis of fly excreta was performed according to [36]. Fly food was supplemented with 0.5% Bromophenol blue sodium salt (B5525, Sigma). Analyses of excreta (including number, shape, size and dye intensity) were carried out on 25 flies housed in 50 mm Petri dishes (5 flies per dish). Digital images of Petri dishes were obtained using an Epson Perfection 4990 Photo scanner and whole-image background adjustments were applied using Adobe Photoshop. Digital scans were analysed using Volocity 64 software (Improvision) using the protocols described in [36]. Colour (Mean Red, Mean Green and Mean Blue) and size (Pixel Count and Perimeter) values were exported to the R environment (R Development Core Team, 2009) and converted to Hue, Saturation, Lightness values. Dye intensity was calculated as 100 − lightness.

### Statistical Analyses

Statistical analyses were performed using JMP software (version 9.0; SAS Institute). Log rank tests were performed on survival curves. Other data were tested for normality using the Shapiro-Wilk W test on studentised residuals and where appropriate log-transformed. One-way analyses of variance (ANOVA) and planned comparisons of means were made using Tukey-Kramer HSD test.
